# Heat Stress and Lipopolysaccharide Stimulation of Chicken Macrophage-Like Cell Line Activates Expression of Distinct Sets of Genes

**DOI:** 10.1371/journal.pone.0164575

**Published:** 2016-10-13

**Authors:** Anna Slawinska, John C. Hsieh, Carl J. Schmidt, Susan J. Lamont

**Affiliations:** 1 Department of Animal Science, Iowa State University, Ames, Iowa, United States of America; 2 Department of Animal Biochemistry and Biotechnology, UTP University of Science and Technology, Bydgoszcz, Poland; 3 Department of Animal and Food Sciences, University of Delaware, Newark, Delaware, United States of America; Washington State University, UNITED STATES

## Abstract

Acute heat stress requires immediate adjustment of the stressed individual to sudden changes of ambient temperatures. Chickens are particularly sensitive to heat stress due to development of insufficient physiological mechanisms to mitigate its effects. One of the symptoms of heat stress is endotoxemia that results from release of the lipopolysaccharide (LPS) from the guts. Heat-related cytotoxicity is mitigated by the innate immune system, which is comprised mostly of phagocytic cells such as monocytes and macrophages. The objective of this study was to analyze the molecular responses of the chicken macrophage-like HD11 cell line to combined heat stress and lipopolysaccharide treatment *in vitro*. The cells were heat-stressed and then allowed a temperature-recovery period, during which the gene expression was investigated. LPS was added to the cells to mimic the heat-stress-related endotoxemia. Semi high-throughput gene expression analysis was used to study a gene panel comprised of heat shock proteins, stress-related genes, signaling molecules and immune response genes. HD11 cell line responded to heat stress with increased mRNA abundance of the *HSP25*, *HSPA2* and *HSPH1* chaperones as well as *DNAJA4* and *DNAJB6* co-chaperones. The anti-apoptotic gene *BAG3* was also highly up-regulated, providing evidence that the cells expressed pro-survival processes. The immune response of the HD11 cell line to LPS in the heat stress environment (up-regulation of *CCL4*, *CCL5*, *IL1B*, *IL8* and *iNOS*) was higher than in thermoneutral conditions. However, the peak in the transcriptional regulation of the immune genes was after two hours of temperature-recovery. Therefore, we propose the potential influence of the extracellular heat shock proteins not only in mitigating effects of abiotic stress but also in triggering the higher level of the immune responses. Finally, use of correlation networks for the data analysis aided in discovering subtle differences in the gene expression (i.e. the role of the *CASP3* and *CASP9* genes).

## Introduction

Acute heat stress is a condition that requires immediate adjustment of the stressed individual to sudden changes of ambient temperatures. Chicken is a warm-blooded animal, but is devoid of many evolutionary adaptations for efficient thermoregulation such as sweat glands, saliva glands, and a long, moist tongue. Therefore, exposure as short as a two hours to high ambient temperature leads to signs of severe heat stress, including decreased number of leucocytes in blood and increased corticosterone level in chickens [1]. Furthermore, overheating causes a drop in egg production, a decrease of egg [2] and meat [3] quality, reduction of feed intake [4], decrease in the growth rate of birds [5] as well as increased susceptibility to diseases and mortality [6]. Therefore, exposure of poultry to heat stress not only generates economic losses [7], but is a threat to food security and animal welfare.

Physiologically, heat stress can cause multi-organ system failure, including the intestinal tract. The tight junctions between the epithelial cells of the guts lose connectivity due to oxidative stress that follows overheating. As a consequence, permeability of the intestinal membrane increases and the intestinal content starts leaking out of the gut, leading to systemic bacterial infection of the organism and the sudden inflammatory response of the immune system (reviewed by [8]). One of the main toxins that is involved in heat cytotoxicity is lipopolysaccharide (LPS), which is a cell wall component of the Gram-negative bacteria and the endotoxin that triggers immune responses. The first line of defense against endotoxemia is the innate immune system, which is comprised mostly of phagocytic cells such as monocytes and macrophages [9–11]. These cells react to stressors by producing large amounts of chemokines, cytokines and nitric oxide species to attract other immune cells to the inflammation site as well as to directly fight off the pathogens. At the molecular level, heat shock causes intracellular proteins to misfold, potentially leading to damage and degradation of the intracellular protein by ubiquitination in the cellular proteasome. The highly conserved chaperone proteins known as heat shock proteins (HSPs) serve as stabilizing factors, binding and refolding the misfolded proteins (reviewed by [12]).

As a part of adaptation, cells are able to adjust to the changing environmental conditions by modulating their gene expression. This gives sufficient flexibility to return to homeostasis under stress conditions, maintain their viability and functions as well as to adapt to long-term changes [13]. Use of recently developed whole genome technologies gives insight into the molecular responses of cells to external challenges. In chickens, transcriptomic approaches have been applied in studying heat stress with both *in vivo* and *in vitro* models, which allowed identification of stress-related gene expression changes in liver [14–17], testes [18], brain [16], heart [17], muscle [15–17] and hepatocellular carcinoma cell line (LMH) [19]. However, to our knowledge, there has been no attempt to study the molecular responses to heat stress and the accompanying endotoxemia directly in the avian immune system. The objective of the current experiment is to investigate the stress-induced molecular reaction of a chicken macrophage cell line to heat and LPS treatment using an *in vitro* model. In this study we address the following scientific questions: (1) does heat stress related endotoxemia lead to dampening vs. increasing of the immune response in macrophage-like cell line? (2) Which chaperones are specific to macrophage-like cell line in chickens? (3) Does double stimuli skew the LPS signaling pathway? (4) Is stress response manifested through apoptosis?

## Materials and Methods

### HD11 cell culture

The chicken macrophage-like HD11 cell line used in this study was established by transforming bone marrow-derived macrophages with Rous-associated virus 2 (RAV-2) [20]. The cells were maintained in RPMI 1640 media (Gibco, Carlsbad, CA, USA) supplemented with 10% of heat inactivated newborn calf serum (Gibco, Carlsbad, CA, USA), 2mM of GlutaMAX^™^ (200 mM L-alanyl-L-glutamine dipeptide in 0.85% NaCl) (Gibco, Carlsbad, CA, USA), 1mM of sodium pyruvate (Gibco, Carlsbad, CA, USA), 0.1 mM of NEAA (Gibco, Carlsbad, CA, USA), 100u/ml of penicillin and 100 μg/ml of streptomycin (Gibco, Carlsbad, CA, USA) (complete medium). The cells were cultured in 75cm^2^ vented cultured flasks at 41.5°C (body temperature of chickens, which is higher than in mammals) and 5% CO_2_ until they became almost fully confluent. The cells were harvested by collecting supernatants and treating the remaining adherent cells with 0.25% trypsin-EDTA for 1 min (Gibco, Carlsbad, CA, USA) followed by trypsin deactivation with complete medium. The cells were centrifuged for 5 min at 200 x g and resuspended in the complete medium to a concentration of 5x10^6^cells/ml. The cells were then seeded in 6-well plates at the volume of 2ml/well and allowed to acclimate before stimulation.

### Heat stress and lipopolysaccharide stimulation

The HD11 cells were stimulated using heat stress and/or lipopolysaccharide (LPS) based on four treatment groups: (1) heat stress and LPS, (2) heat stress only, (3) LPS only and (4) untreated (control). The experiment was replicated three times, on different days, with each experimental replicate consisting of three biological replicates (n = 9 samples per treatment) for every treatment group and time point. Heat stress was applied by transferring cells from physiological temperature (41.5°C) to a CO_2_ incubator with the temperature set at 45°C for two hours. The heat stress conditions (time and temperature) had been previously optimized using cell viability and expression of *HSP70* as indicators for the cells reacting to heat stress without reduction in viability (data presented in S1 File). The LPS treatment was provided at the same time as the heat stress. LPS derived from *Salmonella enterica* serovar *Typhymurium* (Sigma Aldrich, St. Louis, MO, US) was used at the concentration of 5 μg/ml. After two hours of heat stress and/or LPS treatment, the HD11 cells were returned to the CO_2_ incubator with physiological temperature (41.5°C) to recovery from heat stress for 0, 2, 4 and 8 hours. At each of the 4 recovery points the cells from all treatment groups were harvested for RNA isolation. The overall duration of the experiment was from 2 hours to 10 hours, including heat stress (HS) and temperature-recovery at thermoneutral (TN) conditions. Time points were denoted as follows, and each treatment group was contrasted with the response of cells that had been cultured for the same overall time period under thermoneutral conditions: 2 hours (i.e. 2h HS vs. 2h TN), 4 hours (i.e. 2h HS + 2h TN vs. 4h TN), 6 hours (i.e. 2h HS + 4h TN vs. 6h TN) and 10 hours (i.e. 2h HS + 8h TN vs. 10h TN).

### RNA isolation, cDNA synthesis and pre-amplification

HD11 cells harvest post treatment was performed using 1 minute treatment of 0.25% trypsin-EDTA (Gibco, Carlsbad, CA, USA) for collection of the adherent cells. The cells were re-suspended in RNAlater (Ambion, Carlsbad, CA, USA) and stored short term in 4°C until proceeding with the RNA isolation. The total RNA was isolated using RNAqueous^®^ Total RNA Isolation Kit (Ambion, Carlsbad, CA, USA) followed by the genomic DNA removal with DNA-free^™^ DNA Removal Kit (Ambion, Carlsbad, CA, USA). RNA concentration and purity was assessed using NanoDrop spectrophotometer (Thermo Scientific, Wilmington, DE, USA). RNA was stored at -20°C before it was further process in the gene expression study. Briefly, 250 ng of RNA was reversely transcribed using the Fluidigm Reverse Transcription Master Mix (Fluidigm Corporation, San Francisco, CA, USA), with the following incubation protocol: 25°C for 5 min, 42°C for 30 min and 85°C for 5 min. The cDNA served as a template in the pre-amplification reaction carried out using PreAmp Master Mix (Fluidigm Corporation, San Francisco, CA, USA). Each pre-amplification reaction was carried out at the volume of 5μl and included 1.25 μl of the cDNA, 1 μl of the PreAmp Master Mix and 0.5 μl of the pooled DELTAgene Assay Mix (500 nM). To prepare pooled DELTAgene Assay Mix, 100 μM of each primer pairs used in this study (forward and reverse combined, listed in S1 Table) was pooled and diluted with DNA Suspension Buffer to 200 μl total volume and a final concentration of 500 nM. The thermal profile of the pre-amplification included incubation at 95°C for 2 min followed by 10 cycles of: 95°C for 15 s and 60°C. Exonuclease treatment was applied to remove unincorporated primers by incubating the pre-amplified samples with 8 units of the Exonuclease I (New England Biolabs, UK) at 37°C for 30 min of digestion followed by inactivation of the enzyme at 80°C for 15 min. The pre-amplified and purified cDNA samples were diluted 10x in TE buffer and stored at -20°C until further analyses.

### Gene expression study

The gene expression study was carried out using microfluidic RT-qPCR technology (Fluidigm Corporation, San Francisco, CA, USA), following the manufacturer’s recommendations. The primer design and synthesis was carried out with DELTAgene^™^ Assays pipeline (Fluidigm Corporation, San Francisco, CA, USA). S1 Table presents the gene list and the detailed information on the DELTAgene^™^ Assays used in this study. The panel of 44 target genes and 2 reference genes was developed to analyze the gene expression using two 192x24 Integrated Fluid Circuits (IFCs) (Fluidigm Corporation, San Francisco, CA, USA). Each IFC contained all the samples, 22 target gene assays and 2 reference genes. Prior to qPCR, 1.35 μl of pre-amplified and Exo I treated cDNA was combined with 1.5 μl of the SsoFast^™^ EvaGreen^®^Supermix with Low ROX^™^ (2x) (Bio-Rad) and 0.15 μl of the 192.24 Delta Gene Sample Reagent (Fluidigm Corporation, San Francisco, CA, USA). The DELTAgene^™^ Assays were prepared as 20 μl stock by mixing 1 μl of the DELTAgene^™^ Assays (100uM) with 10 μl of the 2x Assay Loading Reagent and adjusted to 20 μl with DNA suspension buffer (low EDTA TE buffer). The samples, assays and the loading reagents were then pipetted on the IFCs. RX loading station (Fluidigm Corporation, San Francisco, CA, USA) was used to load the fluids into the IFCs’ microfluidic channels. qPCR was performed on Biomark^™^ HD (Fluidigm Corporation, San Francisco, CA, USA) using fast program that consisted of incubation step at 95°C for 60 s followed by 30 cycles: 96°C for 5 s and 60°C for 20 s. The fluorescence emission was recorded after each cycling step. Upon qPCR completion, the melting curves were generated by increasing the temperature from 60 to 95°C, followed by continued fluoresce acquisition.

### Data analysis

Raw qPCR data were analyzed and checked for quality using Real-Time PCR Analysis Software (Fluidigm Corporation, San Francisco, CA, USA). To determine the relative gene expression, ddCt method was used [21]. Delta Ct values were obtained by normalizing the Ct values of the target genes with the geometrical mean of the two reference genes (*H6PD* and *RPL4*). Fold induction of the gene expression was estimated as 2^-ddCt^. Untreated (control) samples were used as calibrators. Multivariate analysis of variance (MANOVA) was conducted to determine statistical differences within and between subjects (genes), implemented in JMP Pro 10.0.2 software (SAS Institute, Cary, NC, USA). Time was considered as “within-subject-factor”, whereas treatments—as “between-subject-factors”. Hierarchical clustering and correlation networks were completed in R 3.1.2. Hierarchical clustering was performed using Manhattan distance and Ward linkage based on the log2 fold change values for each treatment and time point. Generation of the correlation network from this study was done with WGCNA package for R [22] with Spearman correlation threshold of 0.8 determined based on the density plot. Additionally, a mutual information network was generated from LMH transcriptomic data [19] using minet package for R [23] with the Aracne algorithm. The network graphs were exported from R for visualization in Cytoscape 3.2.1 [24]. Network clustering was performed on graphs from this study with the MCODE package within Cytoscape and the “Fluff” option was utilized to reduce the number of unconnected nodes in the network clusters [25]. Genes from this current study were selected from the LMH network along with the nearest neighbor gene one degree away. All analysis were completed with the default settings unless otherwise noted above.

## Results

The dataset generated and analyzed in this study included Ct values for 44 target genes and 2 reference genes determined in total RNA samples isolated from HD11 cell line treated with two-hour heat stress (45°C) and 5 μg/ml LPS. Gene selection was based on: Sun et al., 2015 [19]; Xie et al., 2014 [17]; Wang et al., 2013 [18]; Luo et al., 2014 [16]; KEGG pathway database [26]; Li et al., 2014 [15]; Ciraci et al. 2010 [9]; de Boever et al., 2008 [27]; Yue et al 2010 [28]. The molecular function of the selected genes is presented in Table 1. Among 44 target genes analyzed in this gene expression study, 3 genes (*HSF4*, *HSPB8* and *SERPINH1*) with low quality scores of the melting curves were excluded from the dataset and, therefore, 41 test genes were further subjected to the downstream statistical analyses. S2 File presents qPCR dataset (dCt values).

**Table 1:** Function of the target genes used in the gene expression study.

Gene	Name	Entrez ID	Major function^1^	Ref
**Heat stress factors, proteins and co-chaperones**				
*HSF2*	heat shock transcription factor 2	421724	DNA-binding protein; binds heat shock promoter elements (HSE) and activates transcription	1, 2
*HSF4*	heat shock transcription factor 4	427540	DNA-binding protein that specifically binds heat shock promoter elements (HSE)	1, 2
*HSF5*	heat shock transcription factor family member 5	417471	sequence-specific DNA binding and sequence-specific DNA binding transcription factor activity	1, 2
*HSP25*	heat shock protein 25	428310	Involved in stress resistance and actin organization	1
*HSP90AA1*	heat shock protein 90kDa alpha (cytosolic), class A member 1	423463	Hsp90 (90 kDa heat shock protein) is a molecular chaperone that aids protein folding and quality control for a large number of client proteins	2, 3
*HSPA14*	heat shock 70kDa protein 14	418802	Component of the ribosome-associated complex (RAC), a complex involved in folding or maintaining nascent polypeptides in a folding-competent state	1
*HSPA2*	heat shock 70kDa protein 2	423504	In cooperation with other chaperones, Hsp70s stabilize preexistent proteins against aggregation and mediate the folding of newly translated polypeptides in the cytosol as well as within organelles.	1, 2, 3
*HSPB8*	heat shock 22kDa protein 8	416988	Small heat-shock proteins, Displays temperature-dependent chaperone activity	1, 3
*HSPH1*	heat shock 105kDa/110kDa protein 1	418917	Prevents the aggregation of denatured proteins in cells under severe stress, on which the ATP levels decrease markedly. Inhibits HSPA8/HSC70 ATPase and chaperone activities (By similarity)	4
*DNAJA4*	DnaJ (Hsp40) homolog, subfamily A, member 4	415360	Hsp70s co-chaperone, GO annotations related to this gene include unfolded protein binding and heat shock protein binding	3
*DNAJB6*	DnaJ (Hsp40) homolog, subfamily B, member 6	420448	Hsp70s co-chaperone, has a stimulatory effect on the ATPase activity of HSP70; reduces cellular toxicity and caspase-3 activity; suppresses protein aggregation	1, 3
**Apoptosis and stress response genes**				
*BAG3*	BCL2-associated athanogene 3	423931	Hsp70s co-chaperone, inhibits the chaperone activity of HSP70/HSC70 by promoting substrate release. Has anti-apoptotic activity	3, 4
*CASP1*	caspase 1, apoptosis-related cysteine peptidase	395764	Activator of IL1B, can also promote apoptosis	5
*CASP3*	caspase 3, apoptosis-related cysteine peptidase	395476	Effector caspase—involved in the activation cascade of caspases responsible for apoptosis execution	5
*CASP7*	caspase 7, apoptosis-related cysteine peptidase	423901	Effector caspase—involved in the activation cascade of caspases responsible for apoptosis execution	5
*CASP8*	caspase 8, apoptosis-related cysteine peptidase	395284	Most upstream protease of the activation cascade of caspases responsible for the TNFRSF6/FAS mediated and TNFRSF1A induced cell death	5
*CASP9*	caspase 9, apoptosis-related cysteine peptidase	426970	Involved in the activation cascade of caspases responsible for apoptosis execution	5
*CIRBP*	cold inducible RNA binding protein	425789	Plays a protective role in the genotoxic stress response by stabilizing transcripts of genes involved in cell survival	3
*RB1CC1*	RB1-inducible coiled-coil 1	421116	Interacts with signaling pathways to coordinately regulate cell growth, cell proliferation, apoptosis, autophagy, and cell migration	4
*SERPINH1*	serpin peptidase inhibitor, clade H (heat shock protein 47), member 1, (collagen binding protein 1)	396228	Binds specifically to collagen. Could be involved as a chaperone in the biosynthetic pathway of collagen	1
*TP53*	tumor protein p53	396200	Responds to diverse cellular stresses; induces cell cycle arrest, apoptosis, senescence, DNA repair, or changes in metabolism	5
*UBB*	ubiquitin B	396190	Targets cellular proteins for degradation by the 26S proteosome; involved in the maintenance of chromatin structure, the regulation of gene expression, and the stress response	6
**TLR4 signaling and signal transduction genes**				
*IRAK4*	interleukin-1 receptor-associated kinase 4	417796	Activates NF-kappaB in both the Toll-like receptor (TLR) and T-cell receptor (TCR) signaling pathways	5
*JUN*	jun proto-oncogene	424673	Transcription factor that recognizes and binds to the enhancer heptamer motif 5-TGACGTCA-3	5
*MAPK8IP3*	mitogen-activated protein kinase 8 interacting protein 3	426986	JNK-interacting protein (JIP) group of scaffold proteins	5
*MAPK9*	mitogen-activated protein kinase 9	395983	Involved in various processes such as cell proliferation, differentiation, migration, transformation and apoptosis	5
*MyD88*	myeloid differentiation primary response 88	420420	cytosolic adapter protein that plays a central role in the innate and adaptive immune response	5
*NLRC5*	NLR family, CARD domain containing 5	100857413	Probable regulator of the NF-kappa-B and type I interferon signaling pathways	7
*SMAD6*	SMAD family member 6	374096	Mediator of TGF-beta and BMP anti-inflammatory activity	1
*TGFB2*	transforming growth factor, beta 2	421352	Regulates proliferation, differentiation, adhesion, migration, and other functions in many cell types	1
*TGFB3*	transforming growth factor, beta 3	396438	Involved in embryogenesis and cell differentiation	1
*TLR4*	toll-like receptor 4	417241	Innate immune response to bacterial lipopolysaccharide (LPS)	5
*TRAF6*	TNF receptor-associated factor 6, E3 ubiquitin protein ligase	423163	Mediates signal transduction from members of the TNF receptor superfamily; leads to the activation of NF-kappa-B and JUN	5
**Immune-related genes**				
*CCL4*	chemokine (C-C motif) ligand 4	395468	Monokine with inflammatory and chemokinetic properties	7
*CCL5*	chemokine (C-C motif) ligand 5	417465	Chemoattractant for blood monocytes, memory T-helper cells and eosinophils	7
*CD40*	CD40 molecule, TNF receptor superfamily member 5	395385	Transduces TRAF6- and MAP3K8-mediated signals that activate ERK in macrophages and B cells, leading to induction of immunoglobulin secretion	5
*IFNB*	interferon, beta 1, fibroblast	554219	Has antiviral, antibacterial and anticancer activities	5
*IFNG*	interferon, gamma	396054	Has antiviral activity and immunoregulatory functions	5
*IL1B*	interleukin 1, beta	395196	Produced by activated macrophages, inflammatory responses	5
*IL8*	interleukin 8-like 2	396495	Chemotactic factor, inflammatory responses	5
*IL12B*	interleukin 12B	404671	expressed by activated macrophages that serve as an essential inducer of Th1 cells development	5
*IL18*	interleukin 18 (interferon-gamma-inducing factor)	395312	proinflammatory cytokine that augments natural killer cell activity in spleen cells, and stimulates interferon gamma production in T-helper type I cells	5
*iNOS*	nitric oxide synthase 2, inducible	395807	Produces nitric oxide (NO) which is a messenger molecule with diverse functions throughout the body	5
*LITAF*	lipopolysaccharide-induced TNF factor	374125	DNA-binding protein that can mediate the TNF-alpha expression	5
**Reference genes**				
*H6PD*	hexose-6-phosphate dehydrogenase (glucose 1-dehydrogenase)	428188	Oxidizes glucose-6-phosphate and glucose, as well as other hexose-6-phosphates	8
*RPL4*	ribosomal protein L4	415551	Belongs to the L4E family of ribosomal proteins	9

Gene function: GeneCards;

### Relative gene expression

**Fig 1:**
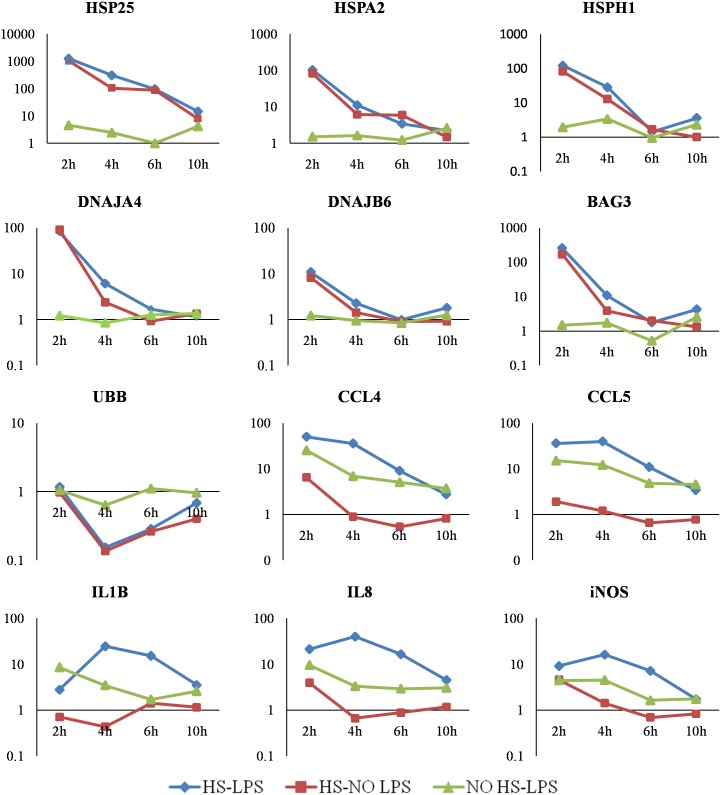
Temporal changes in the relative expression of the genes showing the highest up- or down-regulation upon heat stress and LPS treatment. Fold induction in the relative gene expression was calculated using ddCt method (n = 9). The genes were organized by their function in: heat stress response (*HSP25*, *HSPA2*, *HSPH1*, *DNAJA4* and *DNAJB6*), stress response (*BAG3* and *UBB*) and immune response (*CCL4*, *CCL5*, *IL1B*, *IL8* and *iNOS*). X-axis presents a time point (2h, 4h, 6h and 10h). Y-axis shows fold induction of the gene expression (logarithmic scale). Untreated (control) samples calibrated at the value of fold induction = 1.

The overview of the relative gene expression of all the genes analyzed in this study is presented in S1 Fig. Selected genes, with major changes in mRNA level across all the time points, are shown in Fig 1. Briefly, main changes in the relative gene expression were observed directly post two-hour heat stress and LPS treatment (2h). At this time point, multiple genes related to the heat stress response (*HSP25*, *HSPA2*, *HSPH1*, *DNAJA4* and *DNAJB6*), stress response (*BAG3* and *UBB*) and immune response (*CCL4*, *CCL5*, *IL1B*, *IL8* and *iNOS*) showed high values of expression regulation, which gradually decreased at later time points until reaching baseline at 10 hours post stimulation (10h). The heat stress and LPS treatments applied together had additive effects on the immune-related gene expression (*CCL4*, *CCL5*, *IL1B*, *IL8* and *iNOS*), which peaked at 2h (*CCL4*) or 4h (*CCL5*, *IL1B*, *IL8* and *iNOS*) time point. Another subset of genes showed moderate to low regulation upon heat and LPS treatment and were further analyzed with MANOVA, hierarchical clustering and correlation network methods.

### MANOVA

Complete results of MANOVA are presented as S3 File. Between-subjects tests showed that there was a significant difference between the samples overall (All), mean (Intercept), and slope (Treatment). As for within-subject tests, firstly, sphericity test was significant, so there was no need to use the univariate versions of the statistics. Secondly, Time F-test showed that there were significant differences in fold change between the time points. Finally, Time*Treatment Wilks' Lambda and Pillai's Trace both showed significance of <0.02, which confirmed that both treatment and time caused significant differences in fold changes.

### Hierarchical clustering

**Fig 2:**
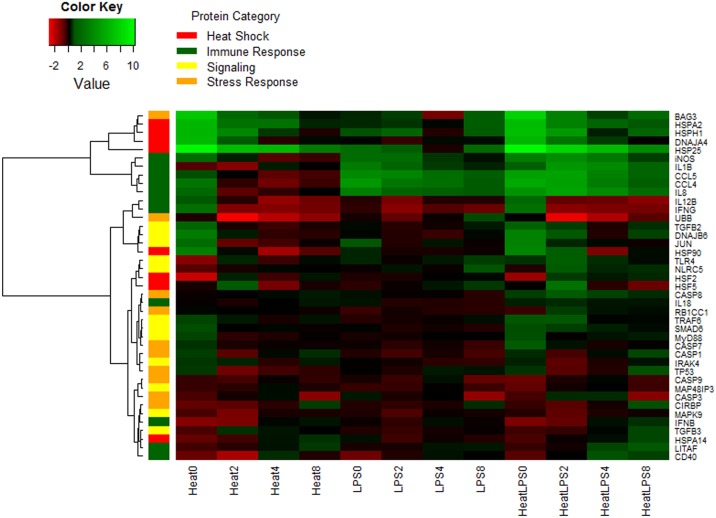
Log_2_ Fold Change Heat Map. A heat map for the 3 different treatments across the 4 time points. Log_2_ fold change calculate based on delta Ct value compared to the control samples and green implies increased expression while red implies decreased expression. Genes on the right are clustered using a hierarchical clustering method and 8 clusters were found. Cluster 1 = *BAG3*, *HSPA2*, *HSPH1*, *DNAJA4*, *HSP25*; Cluster 2 = *iNOS*, *IL1B*, *CCL5*, *CCl4*, *IL8*; Cluster 3 = *IL12B*, *IFNG*, *UBB*; Cluster 4 = *TGFB2*, *DNAJB6*, *JUN*, *HSP90*; Cluster 5 = *TLR4*, *NLRC5*, *HSF2*, *HSF5*; Cluster 6 = *CASP8*, *IL18*, *RB1CC1*, *TRAF6*, *SMAD6*, *MyD88*, *CASP7*, *CASP1*, *IRAK4*, *TP53*; Cluster 7 = *CASP9*, *MAP48IP3*, *CASP3*; Cluster 8 = *CIRBP*, *MAPK9*, *IFNB*, *TGFB3*, *HSPA14*, *LITAF*, CD40. Each gene is color coded based on their major functional category: heat stress response (red), immune response (green), signaling (yellow), and stress response (orange).

Hierarchical clustering of the genes representing similar expression pattern identified 8 clusters, presented on the heat map in Fig 2. It showed a strong cluster of heat shock proteins in the first cluster, where *HSPH1* and *BAG3* were identified to have similar expression pattern across all treatments as *HSPA2*, *DNAJA4*, and *HSP25*. A second cluster grouped the chemokine genes (*IL1B*, *CCL5*, *CCl4* and *IL8*) together with *iNOS*, indicating their induction by LPS in thermoneutral conditions and expression reinforcement by the synergistic combination of LPS and heat stress. The third cluster included two cytokines (*IL12B*, *IFNG*) with *UBB* gene, that were slightly induced by heat stress and heat stress with LPS at 2h time point and then down-regulated at later time points. The remaining five clusters grouped the genes with lower expression, the majority of which belonged to the TLR4 signaling, stress response and apoptosis pathways. Their expression was regulated on much lower level by the applied treatments. Briefly, heat shock factors (HSFs) were classified in the fifth cluster together with TLR4 gene, expressing first down-regulation at the 2h time point followed by slight up-regulation at later time points of heat stress and heat stress combined with LPS treatments. Clusters six and seven grouped all caspase genes and TLR pathway signaling molecules, where were moderately down-regulated. The last cluster, consisting of *CIRBP*, *MAPK9*, *IFNB*, *TGFB3*, *HSPA14*, *LITAF* and *CD40* genes was initially down-regulated by all treatments at 2h, moving towards baseline or slight up-regulation at later time points.

### Network analysis

**Fig 3:**
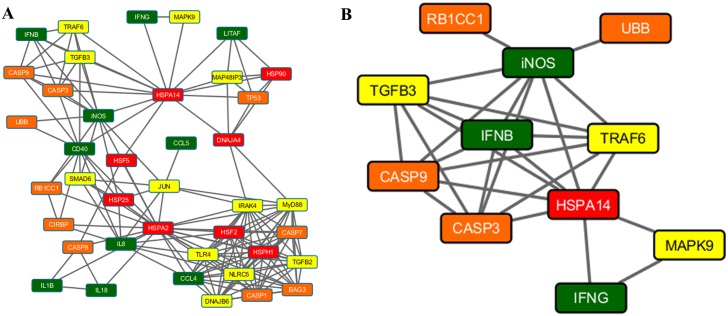
Spearman correlation network for samples treated with at time point 2h. (A) The complete network showing interaction between genes at 0 hours after heat stress treatment. Highly connected hubs includes *iNOS*, *IL8*, *CD40*, and *HSPA14*. (B) Cluster 2 from MCODE network clustering algorithm showing the interaction between *iNOS*, *IFNB*, *CASP9*, *CASP3*, *HSPA14*, *IFNG*, and *MAPK9*. The cluster shows interaction between multiple mechanisms during response to heat stress.

**Fig 4:**
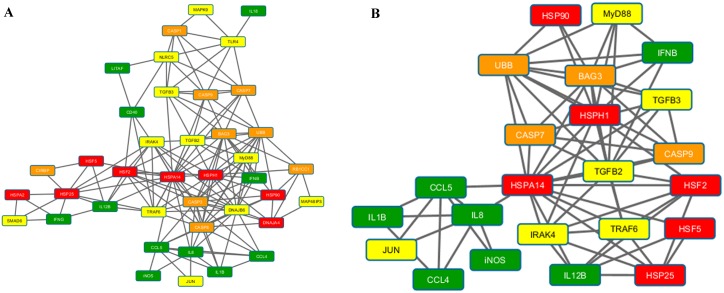
Spearman correlation network for samples treated with LPS at time point 2h. (A) The complete network showing interaction between genes at 0 hours after LPS treatment. Highly connected hubs includes *TLR4*, *IFNB*, *MyD88*, *IL8*, and *HSPA14*. (B) Cluster 1 from MCODE network clustering algorithm showing the interaction between *IL8*, *IL1B*, *CCL5*, *CCL4*, *iNOS*, *JUN*, and HSPA14 after ignoring the hairball to the right of *HSPA14*. The cluster shows interaction between immune response genes from LPS stimulation.

**Fig 5:**
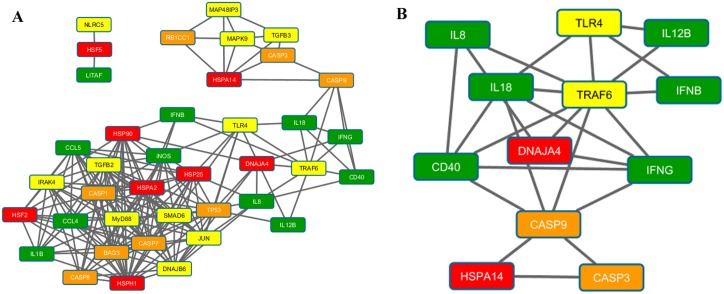
Spearman correlation network for samples treated with LPS and heat stress samples at time point 2h. (A) The complete network showing interaction between genes at 0 hours after LPS under heat stress treatment. Highly connected hubs includes *CASP9*, *IL18*, *IFNB*, *IL8*, and *iNOS*. There appears to be a small cluster of genes related to apoptotic pathway formed near the top of the network. (B) Cluster 2 from MCODE network clustering algorithm showing the interaction between *IL8*, *IL18*, *TLR4*, *TRAF6*, *CD40*, *IFNB*, *IFNG*, and *CASP9*. The cluster shows interaction between multiple mechanisms during response to LPS stimulation under heat stress. Interesting to note once again the additional interaction with the *CASP9*/*CASP3* apoptotic pathway.

Figs 3–5 present the results of the Spearman correlation network for heat stress, LPS and heat stress with LPS treatments at 2h time point, respectively. The overall network of the gene regulation in HD11 cell line treated with heat stress shows *iNOS*, *IL8*, *CD40*, and *HSPA14* as highly connected nodes (Fig 3A). The second cluster from MCODE network clustering identifies a complex interaction involving heat shock (*HSPA14*), immune response (*iNOS*, *IFNB*, and *IFNG*), signaling (*TGFB3*, *TRAF6*, and *MAPK9*), and stress response genes (*RB1CC1*, *UBB*, *CASP9*, and *CASP3*) as shown in Fig 3B. In LPS treatment, the overall network shows *TLR4*, *MyD88*, and *HSPA14* in addition to *IFNB* and *IL8* as important network hubs for the cellular response to LPS stimulation (Fig 4A). Part of the first cluster from MCODE network clustering shows the interaction between five immune response genes identified from previous results with the addition of the signaling gene *JUN* (Fig 4B). Finally, *CASP9* emerged as a hub in the overall correlation network for cellular response to both heat stress and LPS stimulation, leading to a cluster of the genes associated with apoptosis (Fig 5A). Other important hubs identified in the overall network were the immune response genes *IL18*, *IFNG*, *IL8*, and *iNOS*. The second cluster from MCODE network clustering highlighted the interaction of *CASP9* with various immune response genes (Fig 5B). S2 Fig presents the results of the Aracne mutual information network generated from LMH transcriptomic data. There is a large interaction network involving heat shock (*HSPA2*, *HSPA14*, *HSP25* and *DNAJA4*) and signaling (*DNAJB6* and *SMAD6*) genes identified from the differentially expressed genes reacting to heat stress on the LMH cells that were also examined for this current study.

## Discussion

The mechanisms of the molecular responses to heat stress in animals are still not fully understood. One of the consequences of the heat exposure is reduction of the intestinal integrity that leads to increased level of circulating endotoxins and systemic inflammation [29]. Monocytes and macrophages are the key immune cells populations that take part in the innate responses against infection. They recognize bacterial pathogen-associated molecular patterns (PAMPs) through pattern recognition receptors (PRR) located on the cell surface. Activation of the receptors, for example Toll-like receptor 4 (TLR4) by LPS, induces signal transduction to the nucleus and regulation of the cytokine and chemokine expression. Thus, the population of monocytes/macrophages is the first line of defense not only against pathogen invasion, but also endotoxemia resulting from heat stress. In this study we used an *in vitro* model of the chicken monocyte/macrophage cell line (HD11) to examine its molecular responses to acute heat stress and endotoxemia. To create a model of molecular response, we analyzed the transcriptional gene regulation in four gene panels: (1) heat stress factors, proteins and co-chaperons; (2) apoptosis and stress response genes; (3) TLR4 signaling and signal transduction genes and (4) immune-related genes.

The acute heat stress in the current study increased mRNA level of the chaperone genes (*HSP25*, *HSPA2* and *HSPH1*) and their co-chaperones (*DNAJA4* and *DNAJB6*) in HD11 cell line. The cellular responses to stress, including heat stress, results in irreversible aggregation of the proteins in the cytoplasm. The function of the molecular chaperones is to bind and sequester unfolded proteins, including *de novo* protein folding and refolding of nonnative proteins (reviewed by [30]). In our study, expression of molecular chaperones was not induced by LPS alone, which is in agreement with the study performed on rodent splenocytes [31]. Within the timeframe studied (2h post-treatment and later), the highest mRNA abundance was detected for *HSP25* (fold change > 1000). Small heat shock proteins (sHSPs) are the most widespread but the least conserved chaperones that belong to the α-crystallin family. They are ATP-independent chaperones that bind large numbers of unfolded proteins. In case of massive unfolding, they are able to form aggregates and require presence of HSP70 for complete refolding of the proteins (reviewed by [30]). Highly conserved chaperone genes, *HSPA2* and *HSPH1*, were also up-regulated in our study (fold change ~100). Both of the encoded proteins are ATP-dependent chaperones, HSP70 and HSP110, respectively, which are expressed in both constitutive and stress-induced forms. The inducible heat shock proteins are responsible for gaining thermotolerance, whereas the constitutive ones improve stability of the proteins and *de novo* folding even at physiological temperatures [32, 33]. Furthermore, we determined up-regulation of its associated co-chaperones, *DNAJA4* (fold change ~80) and *DNAJB6* (fold change ~10). The function of the DnaJ proteins is to activate ATP hydrolysis and binding Hsc70 (constitutive form) to the aggregated protein [34]. HSP110 combined with HSP70 into an HSP110/HSP70 bichaperone and supported by DnaJ activity, creates a complex that can unfold even stably misfolded and aggregated proteins [35].

In our study, gene regulation of the molecular chaperones and co-chaperones was the highest immediately after two-hours of thermal stimulation of HD11 cells and it decreased during temperature-recovery time. These results are in agreement with previous studies, based on *in vitro* and *in vivo* models. High up-regulation of *HSP25*, *DNAJA4*, *HSP70* (*HSPA2*) and slightly lower of *HSP90AA1* was previously determined in testes of roosters treated with acute heat stress [18]. Also, expression of *HSP25* and *HSPH1* was increased in brain, liver and leg muscle of the broiler chickens subjected to acute heat stress [16]. Furthermore, *HSP25*, *HSPA2*, *HSPH1* and *DNAJB6* gene expression was also enriched in the chicken male white-leghorn hepatocellular (LMH) cell line stimulated with an acute heat stress [19]. An interaction network involving these enriched gene expression function is suggested by our network analysis (S2 Fig). In contrast, expression of the heat shock transcription factors (HSFs) such as *HSP2*, *HSP3* and *HSP4*, was up-regulated in broiler chickens subjected to heat stress [17] but not in the LMH cell line [19]. In our study, mRNA abundance of *HSF2* and *HSF5* was only slightly increased at two hours of temperature-recovery time. Even though HSFs regulate the transcription of HSPs, both *HSF2* and *HSF5* seem to be poorly induced by the heat stress. Activation of *HSF2* is not enhanced by heat stress directly, but upon accumulation of the protein due to decreased ubiquitination, modulated during stress and development [36]. Therefore, we hypothesize that the slight increase in expression of those genes may be related with the decreased expression of ubiquitin B gene (*UBB*). Also, there must be an alternate mechanism involved of the HSPs activation (e.g. via *HSF1*).

One of the pathological effects of a heat related stressor is induction of apoptosis [37]. To analyze the hypothesis that heat stress activates programmed death of HD11 cells upon heat stress, we evaluated mRNA abundance in a panel of apoptosis-related and stress- response genes, including pro-apoptotic (e.g. *CASP* gene family, *TP53*) and anti-apoptotic (e.g. *BAG3*, *CIRBP*, *HSPA2*) regulators. As a result, there was little to no regulation of the major apoptosis genes, including *TP53* and highly elevated expression of anti-apoptotic gene *BAG3* in cells stimulated with heat stress (fold change ~ 80) and heat stress together with LPS (fold change ~ 120). *BAG3* belongs to the family of co-chaperones that interact with the ATPase domain of the HSP70 protein family through a specific domain called BAG domain. The expression of *BAG3* is constitutive in monocytes, but is induced by different stressors, such as high temperature [38]. BAG3 protein has strong anti-apoptotic and pro-survival activity. The proposed underlying mechanisms (reviewed by [39]) include promoting of HSC70/HSP70 chaperoning activity of anti-apoptotic factors as well as negative regulation of the proteins delivery to proteasome (through binding of ubiquitin-like domain in HSP70). A dramatic increase in expression of *BAG3* was also demonstrated in testes [18], brain, liver and leg muscle [16] of heat stressed chickens, suggesting an important role in mitigating heat stress effects at the molecular level.

Even though we did not observe effects of cell death nor increased expression of the apoptosis genes in our study, the network analysis showed that *CASP3* and *CASP9* genes were located centrally in the nodes of the Spearman correlation networks in the HD11 cells treated with heat stress (Fig 3) and heat stress with LPS (Fig 5). Furthermore, the expression of *CASP8* was slightly up-regulated in the cells treated with both stimuli. Recent reports show that the endothelial-cell apoptosis was induced in the HUVEC cells upon heat stress through mitochondrial (intrinsic) pathway [37, 40]. Gu et al. (2014) reported that the subset of p53 protein (major regulator of the apoptosis) was translocated to the mitochondria, where it activated caspase-3 and therefore triggered a first, transcription-independent, wave of apoptosis [37]. However, it has been proved that thermal activation of *HSP70*, *HSP90* and *HSP27* in human colon cancer cell lines reduced effects of induced apoptosis [41]. Finally, it has been indicated that the heat stress contributed to the death of the rat neuron cells *in vitro*, through caspase-3 activation, but these effects were delayed by caspase inhibitors until 10 hours of the temperature-recovery time [42]. These results indicate that there is potential interaction between HSPs and caspases in the HD11 cell line, however a different experimental approach should be taken to verify this (e.g. longer recovery time).

The response of the HD11 cell line to LPS treatment was up-regulation of the pro-inflammatory cytokines (*IL1B*) and chemokines (*CCL4*, *CCL5* and *IL8*) as well as increased transcription of inducible nitric oxide synthase (*iNOS*). Such a transcriptional pattern reflects the typical molecular mechanisms of the immune response expressed by monocytes and macrophages upon activation with LPS [9]. However, the heat stress stimuli applied together with LPS synergistically increased the expression of those genes, particularly at the 4h time point. The mechanism by which heat stress modulates immunity is in part due to the extent of induction of heat shock proteins and their immunogenic properties, especially in relation to antigen presenting cells (APCs), such as macrophages, which may present HSP70 through endogenous route and MHC I (i.e. cross-priming) (reviewed by [43]). HSP70 has also been reported to activate monocytes [44]. Extracellular HSPs are considered danger-associated molecular patterns (DAMPs) that activate the danger signaling cascade [45–47]. HSP70 can be actively secreted from the macrophages [48], and this process is enhanced by exposure of the cells to *E*. *coli*, LPS, bacterial proteins and heat stress [49,50]. Furthermore, extracellular HSPs can interact with the receptors on APCs (e.g. CD14 or CD91) and as such they can stimulate immunoregulation [49]. In our study, the HD11 cell line was exposed to the heat stress and LPS stimulation delivered at the same time, which triggered vast changes in the mRNA expression of HSP (including *HSPA2* that encodes HSP70) genes directly after heat stimulation. Two hours later, the expression of the immune genes increased in the cells stimulated with both LPS and heat stress. This suggests that the immune response to LPS of HD11 cell line has been enhanced by application of heat stress.

## Conclusions

In this study, we evaluated response of a chicken macrophage-like (HD11) cell line to heat stress and LPS stimulation. This *in vitro* model was aimed to mimic the responses of the innate immune system to elevated temperatures and the resulting endotoxemia. The monocyte/macrophage cell line responded to heat stress with dramatically increased mRNA abundance of the *HSP25*, *HSPA2* and *HSPH1* chaperones as well as *DNAJA4* and *DNAJB6* co-chaperones. The anti-apoptotic gene *BAG3* was also highly up-regulated, providing evidence that the cells expressed pro-survival processes. The immune response of the HD11 cell line to LPS in the heat stress environment (up-regulation of *CCL4*, *CCL5*, *IL1B*, *IL8* and *iNOS*) was higher than in thermoneutral conditions. Therefore, we propose the potential influence of the extracellular HSPs not only in mitigating effects of abiotic stress but also in triggering the higher level of the immune responses. The approach of analyzing the expression of the targeted panels of genes, selected from prior RNAseq experiments proved to be a useful tool, especially in combination with high-throughput qPCR. Finally, use of correlation networks for the data analysis aided in discovering subtle differences in the gene expression (i.e. the role of the *CASP3* and *CASP9* genes). The fundamental insights gained in this study into the molecular genetic response of avian innate immune cells to heat stress and endotoxemia pave the way toward genetic improvement of heat-resilience in chicken populations, after future studies of natural genetic variants in these identified genes.

## Supporting Information

S1 Fig Log2 fold change in the gene expression induced by treatments. (TIF)

S2 Fig Aracne mutual information network generated from LMH transcriptomic data. (TIF)

S1 File Two-step optimization of heat stress conditions for HD11 cell line. (PDF)

S2 File qPCR dataset (dCt values). (XLSX)

S3 File Overview of MANOVA results. (XLSX)

S1 Table DELTAgene^™^ Assays used in gene expression study. (PDF)
