# Supplementary figures and images for: Heat Stress and Lipopolysaccharide Stimulation of Chicken Macrophage-Like Cell Line Activates Expression of Distinct Sets of Genes
Source: PLoS One. 2016 Oct 13;11(10):e0164575. doi: 10.1371/journal.pone.0164575 (PMC5063343; doi:10.1371/journal.pone.0164575)

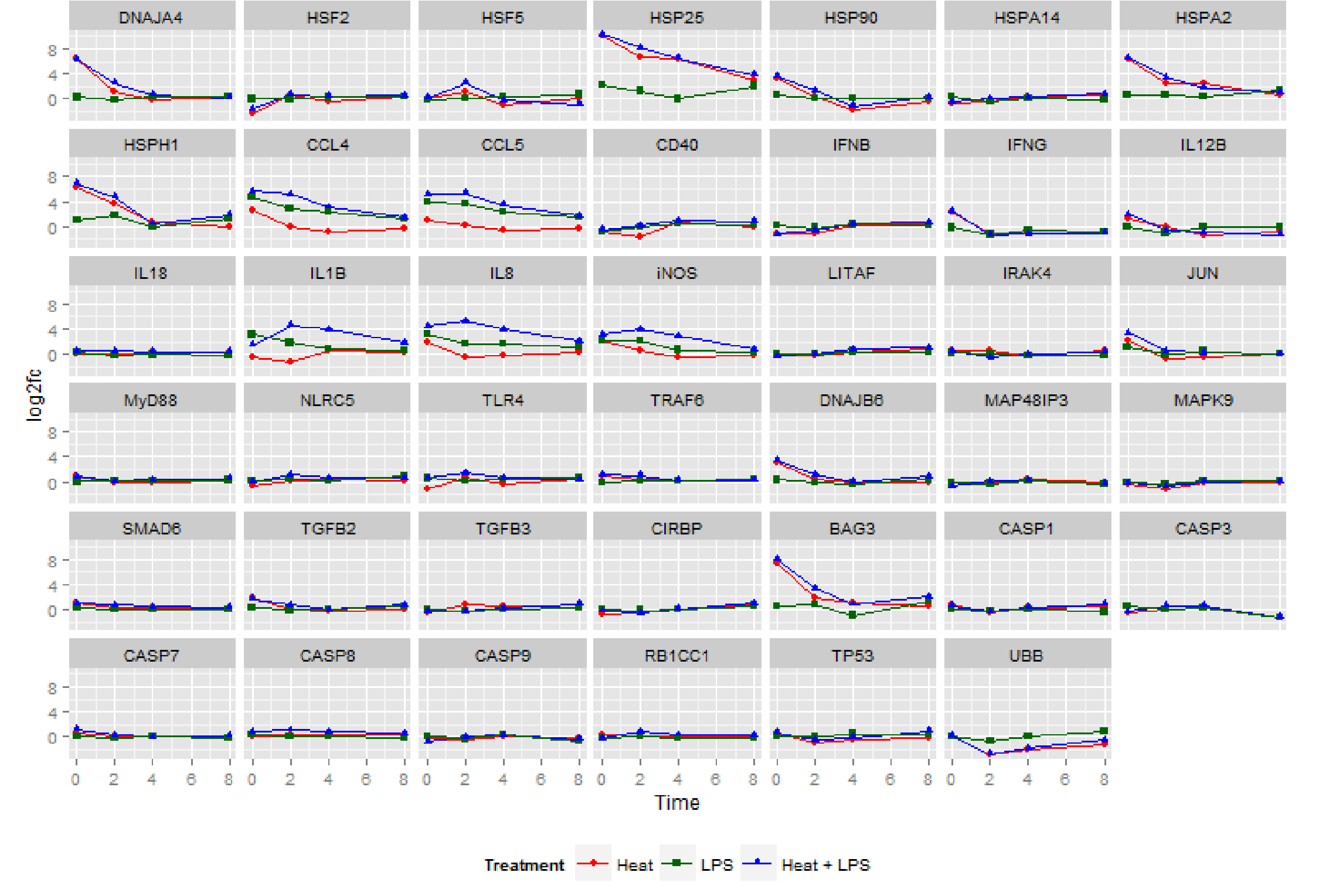

Supplement: S1 Fig — (TIF) [file pone.0164575.s001.tif]

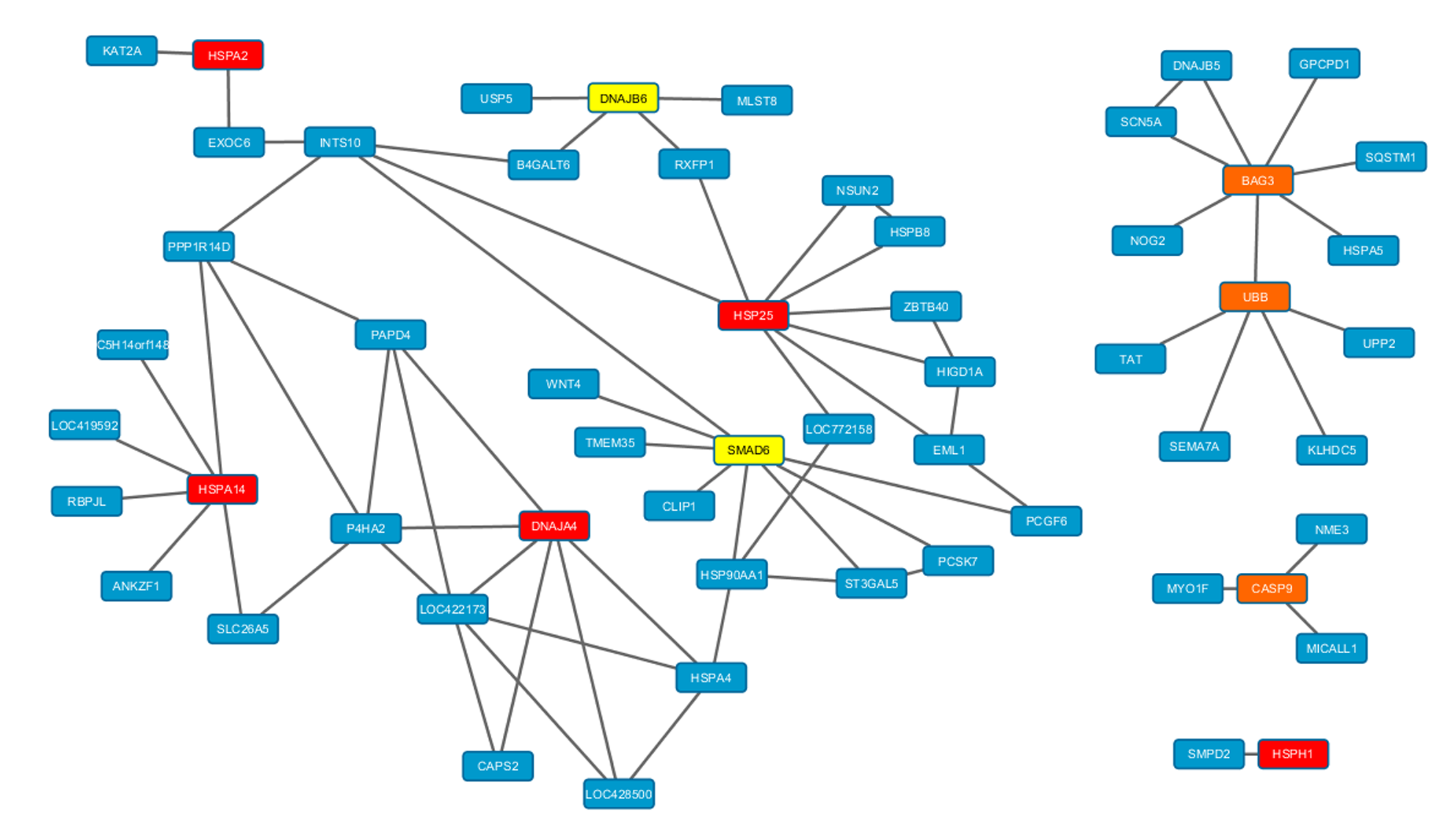

Supplement: S2 Fig — (TIF) [file pone.0164575.s002.tif]
