# Supplementary material for: Heat Stress and Lipopolysaccharide Stimulation of Chicken Macrophage-Like Cell Line Activates Expression of Distinct Sets of Genes
Source: PLoS One. 2016 Oct 13;11(10):e0164575. doi: 10.1371/journal.pone.0164575 (PMC5063343; doi:10.1371/journal.pone.0164575)
Supplement: S1 File — (PDF) [file pone.0164575.s003.pdf]

S1 File

Two-step optimization of heat stress conditions for HD11 cell line.

1. Selection of temperature: 45°C vs. 47°C (41.5°C was considered thermoneutral). Pilot study has been designed to determine the conditions of heating that would be considered a stress to the cells but without detrimental effects on their viability. Trypan blue exclusion assay was used to assess viability:

Table 1. Viability of HD11 cells after incubation in different thermal conditions

Temperature/Time	1 hour	3 hours
41.5°C	86 %	84 %
45°C	83 %	85 %
47°C	69 %	37 %

Results: Temperature of 45°C did not reduce viability of the cells when compared to cells kept in thermoneutral conditions. The temperature of 47°C was considered detrimental and as such, removed from the study.

2. Selection of heat stress duration (1hr vs. 3 hrs.) at 45°C (41.5°C was considered thermoneutral control). Gene expression regulation of HSP70 (by RT-qPCR) was indicative of molecular responses to heat stress

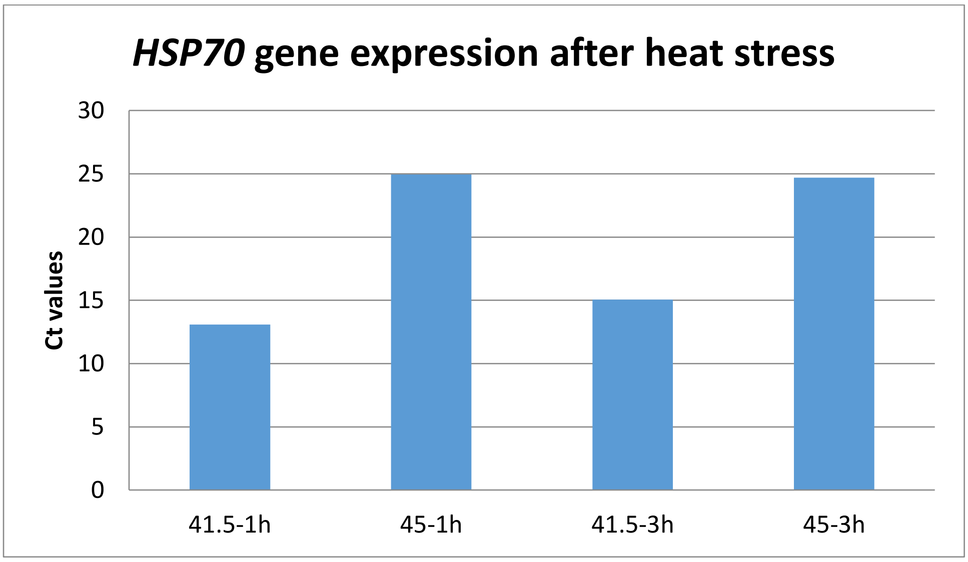

Results: There were no differences between HD11 cells incubated for 1hr vs. 3hrs. at elevated ambient temperature (45°C). For further experiments, intermediate duration of heat stress (i.e. 2hrs) was selected.
