# Supplementary material for: Heat Stress and Lipopolysaccharide Stimulation of Chicken Macrophage-Like Cell Line Activates Expression of Distinct Sets of Genes
Source: PLoS One. 2016 Oct 13;11(10):e0164575. doi: 10.1371/journal.pone.0164575 (PMC5063343; doi:10.1371/journal.pone.0164575)
Supplement: S1 Table — (PDF) [file pone.0164575.s006.pdf]

S1 Table. DELTAgene™ Assays used in gene expression study

Gene	Entrez ID	Forward primer	Reverse primer
BAG3	423931	ACCACAACAGCCGAACCA	GATGGGCCATTTGCTGATGAC
CASP1	395764	GTGGAGTGCCATGAAGACAAA	AGGTGCTGTCAGAGGTCAAA
CASP3	395476	CGCTCAGGGGAAGATGTATCA	CCAGAGTCCACAGACTTGCTA
CASP7	423901	ATGACCGAAGCTGTGAGGATA	GGCAAAACAAGCAGCATCAC
CASP8	395284	GCTTGTAACAGAGGGGCAAA	CACAGATGATGCCAGCCAAA
CASP9	426970	TTTCAGGTCCCTGTGCTTCC	TTCCGCAGCTCCACATCAA
CCL4	395468	CCTCATCCAGAGGCACTACA	GCTTGACGCTCTGCAGGTA
CCL5	417465	CTGCCCCAGAATCATGTGAA	CAGCTCCAGGAAGTTGATGTA
CD40	395385	AGCCTGGTGATGCTGTGAA	CTCACAGGGTGTGCAGACA
CIRBP	425789	TCGGCTTTGTGACGTTTGAA	CTCTGATCTGACGGCCATCTA
DNAJA4	415360	AAGTACCACCCCGACAAGAA	TTGGGTCCGACAGAACTTCA
DNAJB6	420448	GTGGATTCCCTGCTTTTGGAA	CTCCATGTCCCAGAGAACCA
H6PD	428188	ATGTACCGGGTGGACCACTA	AACTGACGGTTCTGATCTCGAAA
HSF2	421724	AGGACTTCCAGGCCATGTTA	CATCTGCACGGAGCTTGTAA
HSF4	427540	TTCTGTTTGGCCAGCTCCAA	ACAACCCACTGTCAAGCATCA
HSF5	417471	AGTCCTACAGCTCTGACCTACA	GCAGGAACTCAACCGCATAC
HSP25	428310	GCACGCAGAGACCATCTTCA	TGCTCGAAGCTGCTCATGAA
HSP90AAI	423463	ACACATGCCAACCGCATTTA	CCTCCTCAGCAGCAGTATCA
HSPA14	418802	TTGGCGGAGTGTACTTCACA	CCTGGGATTTCCTCTGATGTCA
HSPA2	423504	CCACCATTCCCACCAAACAA	ATACACCTGGACGAGGACAC
HSPB8	416988	GAGCTGACGGTCAAAACCAA	GAGACGATGCCTCCTTCCA
HSPH1	418917	GTAGTTTCGTTCGGCTCCAA	CTGTGTTGTGGGCATGAGTAA
IFNA	396398	CCACACCTTCCTCCAAGACAA	TTGTGGATGTGCAGGAACCA
IFNB	554219	GCTCACCTCAGCATCAACAA	TCCCAGGTACAAGCACTGTA
IL12B	404671	CTTCTGGAAGCACAGTGGAAAC	AGCTGGTGTCTCATCGTTCC
IL18	395312	CGTGGCAGCTTTTGAAGATGTA	CTGAATGCAACAGGCATCCC
IL1B	395196	TGCTTCGTGCTGGAGTCAC	GGCATCTGCCCAGTTCCA
IL8	396495	CCCCACTGCAAGAATGTTGAAA	GTGCCTTTACGATCAGCTGTAC
iNOS	395807	GGACCGAGCTGTTGTAGAGATA	AGCAGCTGAGTGATGATCCA
IRAK4	417796	CAATGGTTCGCTGCTTGACA	CGCAGTACCTTGAGCAATTTCA
JUN	424673	TCCCCTGTCCCCTATTGACA	CGCCGCAATTCTGTTTCTCA
LITAF	374125	ATCGTGACACGTCTCTGCTA	AGCATCAACGCAAAAGGGAA
MAPK8IP3	426986	GCCAAAGCCAAAATGGAAACC	GTCGAGCAACAATCGCTTCA
MAPK9	395983	TTACAGAGCGCCAGAGGTTA	TCTCCCATGATGCAACCAAC
MyD88	420420	GAACGTGTGTGTGGTCCATTA	TGAAATGACGACCACCATCC
NLRC5	100857413	TTTGCTGCTGCGCTTTCA	TGTGATGCTTCCACCTGTCA
RB1CC1	421116	CAAGCAGGAGCTTGCGAATA	CATCTTGGTCAGCATGAAGCA
RPL4	415551	TTCTGCCTTGGCAGCATCA	AGGAAGTTCTGGGATCTCCTCA
SERPINH1	396228	AACTGATGGAGCCCTTATTGTCA	TCACCATGAAGCCACGGTTA
SMAD6	374096	GTGTGCTGCAATCCGTACC	TTAGGAGACAGCCGGGAGTA
TGFB2	421352	CGTGCTCTAGATGCTGCCTA	GCCAAGATCCCTCTTGAAGTCA
TGFB3	396438	GGGCCCTGGATACCAACTAC	GGTCCTGTCGGAAGTCAATGTA
TLR4	417241	CCTGCTGGCAGGATGCA	TGTTCTGTCCTGTGCATCTGAA
TP53	396200	GCTGAACCCCGACAATGAGATA	GCGCCTCATTGATCTCCTTCA
TRAF6	423163	TGCCCCAGTACCATGCTTTTA	GTGTCGTGCCAGTTCATTCC
UBB	396190	GGGGCGTCGGAGGATATATAA	CACAACAGACCAAACCCGTTA
